# Empirical estimation of marine phytoplankton assemblages in coastal and offshore areas using an *in situ* multi-wavelength excitation fluorometer

**DOI:** 10.1371/journal.pone.0257258

**Published:** 2022-02-03

**Authors:** Taketoshi Kodama, Yukiko Taniuchi, Hiromi Kasai, Tamaha Yamaguchi, Misato Nakae, Yutaka Okumura

**Affiliations:** Fisheries Resources Institute, Japan Fisheries Research and Education Agency, Yokohama, Japan; University of Shiga Prefecture, JAPAN

## Abstract

Phytoplankton assemblages are essential for understanding the quality of primary production in marine ecosystems. Here, we describe the development of a methodology for monitoring marine phytoplankton assemblages using an *in situ* multi-wavelength excitation fluorometer (MEX). The MEX recorded the fluorescence excited with nine light-emitting diodes, temperature, and sensor depth. We prepared reference datasets comprising MEX fluorescence and plant pigment-based phytoplankton assemblages of nine chemotaxonomy groups (diatoms, dinoflagellates, cryptophytes, chlorophytes, haptophytes type 3, haptophytes type 4, prasinophytes, cyanophytes, and prochlorophytes). Conversions from the MEX fluorescence to the phytoplankton assemblages were conducted with two processes. First, target MEX fluorescence was decomposed using a linear inverse model for calculating coefficients. Second, pigment-based chemotaxonomy of the target MEX fluorescence was reconstructed using the coefficients and the chemotaxonomy assemblages of the reference data. Cross-validation analyses indicated good estimation of the proportion of diatoms, dinoflagellates, cryptophytes, cyanophytes, and prochlorophytes with MEX, and when chlorophytes, haptophytes and prasinophytes were summarized as other eukaryotes, the positive correlation was seen between proportions estimated with MEX and pigments as same as other five chemotaxonomy groups. Repeated MEX observations were conducted in the Kuroshio, the Sea of Japan, the Oyashio, and the Okhotsk Sea. The water-column integrated biomass indicated that the diatoms were an important primary producer in the Oyashio and the Okhotsk Sea, while eukaryotes were important in the Sea of Japan and prochlorophytes were important in the Kuroshio. Our method with the MEX will be a powerful tool to understand and estimate the chemotaxonomy-level assemblages and biomass in the ocean.

## Introduction

Phytoplankton biomass and composition are essential parameters for understanding marine ecology [1], with differences in phytoplankton assemblages linked to differences in higher-trophic marine organism assemblages [2]. Additionally, phytoplankton assemblages affect biogeochemical element cycles. For example, diatoms change dissolved Si into particulate Si. Lorrain et al. [3] suggested that diatoms are decreasing in oceans based on trend of carbon stable isotope ratio of tuna, although evidence regarding changes in the phytoplankton community is limited. Therefore, monitoring phytoplankton assemblages is essential at global and local scales, and consistent analysis methods are required [4].

Microscopic observation is the most basic method for monitoring phytoplankton assemblages. Chemotaxomic identification using photosynthesis and photoprotective marker pigments represents an alternative and more convenient method [1]. However, chemical analyses during plant pigment identification using high-performance liquid chromatography (HPLC) require ≥30 min, and plant pigment markers degrade quickly [5], preventing routine and high-resolution observations. Ultra-HPLC required a short run time (7 min) [6], but high-resolution observations of HPLCs are still difficult considering shipboard operations such as water samplings and filtration.

*In situ* multi-excitation chlorophyll fluorimetry using a submersible spectrofluorometer is a promising technique for rapidly evaluating phytoplankton-community structure [7]. Fluoroprobe (bbe Moldaenke, Schwentinental, Germany) is a dominant submersible spectrofluorometer for monitoring phytoplankton communities in the ocean [8]. Fluoroprobe uses five light-emitting diodes (LEDs; operating at 470, 525, 570, 590, and 610 nm) to excite the photosystem II antenna system [8]. It allows detection of the chlorophyll-based biomass of four groups (green, blue, brown, and mixed) [7]. The Multi-Exciter (MEX; JFE-Advantech, Hyogo, Japan) is an alternate submersible spectrofluorometer that uses nine LEDs (375, 400, 420, 435, 470, 505, 525, 570, and 590 nm) to excite the accessory pigments associated with the photosystem II antenna system and can estimate the chlorophyll-based biomass of three groups (green algae, brown algae, and cyanobacteria) as the default [9]. Comparing the chemotaxonomy approaches, submersible spectrofluorometers are conventional and environment-friendly, but the taxonomic resolutions of spectrofluorometers with default programs are not comparable with pigment estimation or microscopic observations. In addition, phytoplankton community structure based on spectrofluorometers was rarely reported in oceanic observations.

Wang et al. [10] indicated that the default conversion method from the fluorescence to the phytoplankton assemblages using the MEX software (MFL software, JFE-Advantech, Hyogo, Japan) is limited in the field studies and that site-specific methods are required for better estimations. The conversion techniques described by Wang et al. [10] were adequate for estimating phytoplankton assemblages from fluorescence measurements in the East China Sea. Wang et al. [10] converted the normalized fluorescence to principal components using a principal component analysis (PCA), make the multi-linear regression model with the principal components (PCA scores) and phytoplankton communities, and then calculate the phytoplankton community structure based on the multi-linear regression model. However, they used software MATLAB (MathWorks, USA) for calculation, and the program code and data sets are not opened: these made the field application of their methods difficult. Therefore, MEX observations are not usually converted to phytoplankton-community structures, as described by Fujiwara et al. [11].

This study developed methods to rapidly evaluate phytoplankton assemblages using an open-source computer program with the MEX. Specifically, we used an empirical method to convert MEX data to phytoplankton compositions. We evaluated this conversion method using cross-validation analyses. Then, we applied this method to field observations in the coastal and offshore areas of the ocean around Japan to evaluate the spatiotemporal variations of phytoplankton assemblages.

## Materials and methods

### Pigment-based plankton assemblages

**Fig 1:**
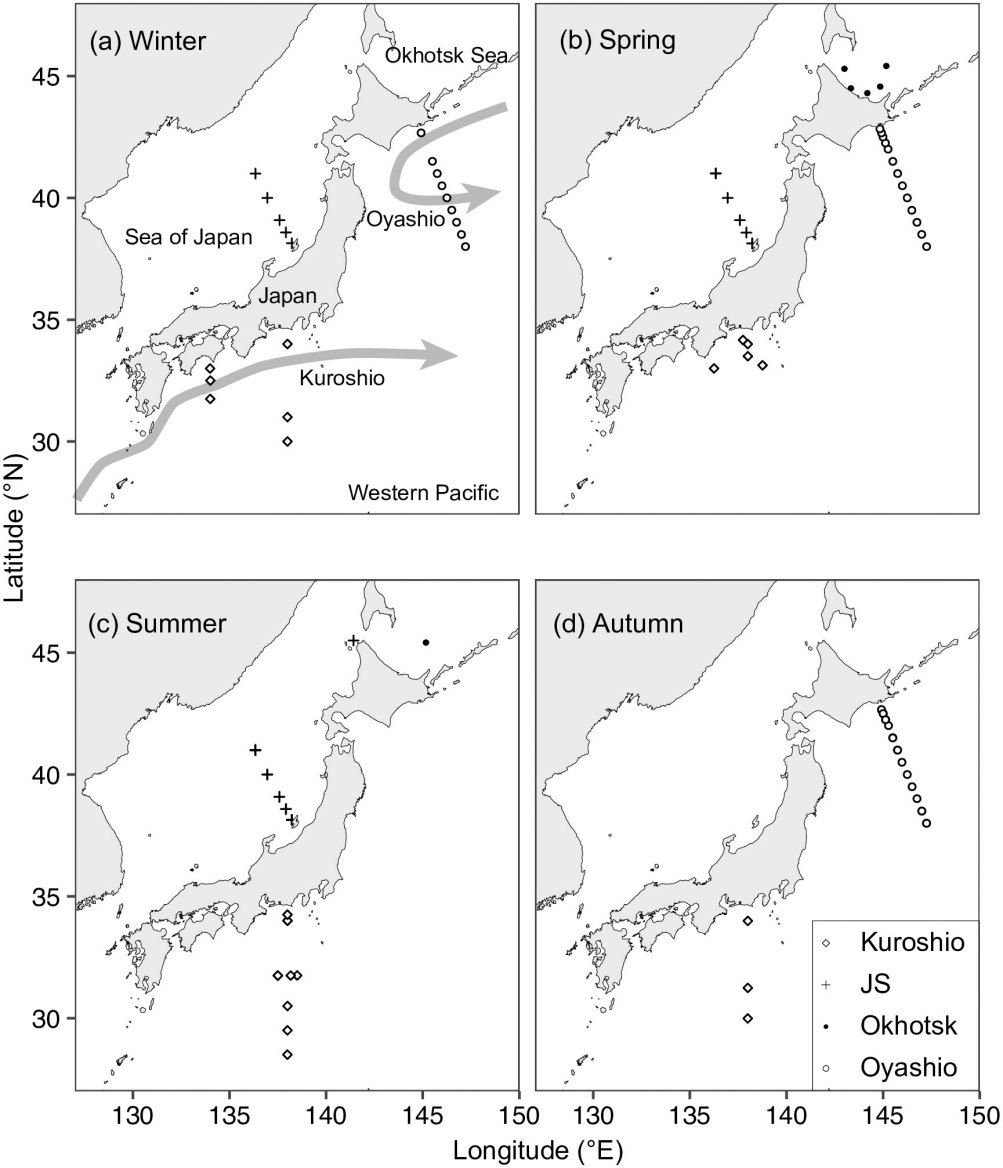
Maps of plant pigment sampling with MEX (Multi-Exciter) observations. Maps show observations in (a) winter, (b) spring, (c) summer, and (d) autumn. Different symbols denote different areas: diamond, Kuroshio; cross, Sea of Japan (JS); closed circle, Okhotsk Sea; and open circle, Oyashio, including the Oyashio–Kuroshio transition. Schematic flows of the Kuroshio and the Oyashio are denoted as arrows. The red points indicated the monitoring stations (A01, C3400, N4, and SI09) of the MEX observations. The map was made with Natural Earth.

The observation areas (28.5–45.5°N, 134–147.25°E, Fig 1) were in the Japanese territorial sea, Japanese EEZ, and high seas; permission for observation was not required except from Japanese government, and all the observations were permitted by the Japan Fisheries Research and Education Agency and Fisheries Agency of Japan.

We collected 271 samples for plant pigment analysis in 2019 and 2020 at 107 stations at depths of between 10 m and 170 m (Fig 1, Table 1). The MEX observations were also conducted at these stations. The observations were divided into four areas based on geographical characteristics: the Kuroshio and Oyashio areas (including the Oyashio–Kuroshio transition area), the Okhotsk Sea (Okhotsk), and the Sea of Japan (JS). Winter, spring, summer, and autumn were defined as occurring from December to February, March to May, June to September, and October to November except in the Okhotsk. Observations in the Kuroshio area were conducted throughout the four seasons, and observations were not conducted in autumn and summer in the JS or Oyashio areas, respectively. In the Okhotsk, observations were only conducted in June (including two stations conducting observations on 31 May 2019) and September in both 2019 and 2020; observations of the Okhotsk area conducted in June were defined as spring observations, and those in September as summer observations in Table 1.

**Table 1:** List of the number of samples used for discrete plant pigment analysis.

	Winter	Spring	Summer	Autumn
Discrete plant pigments				
Kuroshio	27	18	45	13
JS	16	26	48	0
Okhotsk	0	14	1	0
Oyashio	17	13	0	33

Observations of the Okhotsk area conducted in June were considered as spring observations.

Particles in 0.5 L to 2.3 L of seawater were collected on 0.7-μm glass-fiber filters (Whatman GF/F) under gentle suction (<0.02 MPa). The filter was immersed in 1 mL of *N*, *N*-dimethylformamide (DMF) and stored in the dark at <−20°C until onshore analysis or flash-frozen in liquid nitrogen without DMF immersion and stored at <-70°C until onshore analysis. The former storage method can maintain the status of chlorophyll and accessory pigments (except diadinoxanthin) for 180 days [12]. The latter method is the conventional method for plant pigment analysis.

The concentrations of extracted plant pigments were measured using an HPLC system (Shimadzu, Kyoto, Japan). DMF (1 mL) was added to samples stored without DMF. After filter removal, the extracts were centrifuged at a maximum speed of 17,000*g* for 10 min, followed by injection of 300 μL of supernatant into the HPLC system after mixing with 90 μL ultrapure water. Plant pigments were analyzed according to the protocol described by Zapata et al. [13]. During analysis, a 4.6 mm × 150-mm reversed-phase C_8_ column (Symmetry 3.5 μm; Waters, Milford, MA, USA) with a guard column (Symmetry Sentry; Waters) was maintained at 25°C. Plant pigments were detected using a photodiode array UV-Vis detector (SPD-M10AV; Shimadzu). According to reference standards, 20 types of major plant pigments were identified by their retention times and absorption spectra and quantified by the peak areas (Danish Hydraulic Institute, Hørsholm, Denmark). The following 13 types of plant pigments were detected: peridinin, 19’-butanoyloxyfucoxanthin, fucoxanthin, 19’-hexanoyloxyfucoxanthin, neoxanthin, prasinoxanthin, violaxanthin, alloxanthin, lutein, zeaxanthin, chlorophyll *b*, monovinyl chlorophyll-*a* (MVChla), and divinyl chlorophyll *a* (DVChla). The sum of MVChla and DVChla concentrations was described as total chlorophyll-*a* concentration (TChla).

Pigment-based chemotaxonomic phytoplankton assemblages were calculated using a Bayesian compositional linear inverse technique [14] using R software [15]. For the calculation, we used the “limSolve” package [16], which is comparable to the Mackey et al. [1] program [14]. Some plant pigment ratios were previously published to calculate chemotaxonomic phytoplankton compositions using the CHEMTAX program in our study area [17–19]. The initial plant pigment ratios of nine algal classes (diatoms, haptophytes type 3, haptophytes type 4, dinoflagellates, cryptophytes, prasinophytes, chlorophytes, cyanobacteria, and prochlorophytes) were calculated using the initial ratio described in Hashihama et al. [18] (Table 2).

**Table 2:** Ratios of biomarker pigments for the bayesian compositional linear inverse approach based on Hashihama et al. [18].

	Per	But	Fuc	Hex	Pra	Vio	All	Zea	Chlb	MVChla	DVChla
Diatoms	0	0	75	0	0	0	0	0	0	100	0
Haptophytes 3	0	2	12	125	0	0	0	0	0	100	0
Haptophytes 4	0	52	13	65	0	0	0	0	0	100	0
Dinoflagellates	106	0	0	0	0	0	0	0	0	100	0
Cryptophytes	0	0	0	0	0	0	23	0	0	100	0
Prasinophytes	0	0	0	0	15	11	0	0	2	100	0
Chlorophytes	0	0	0	0	0	5	0	1	3	100	0
Cyanophytes	0	0	0	0	0	0	0	34	0	100	0
Prochlorophytes	0	0	0	0	0	0	0	69	114	0	100

Per: peridinin; But: 19’-butanoyloxyfucoxanthin; Fuc: fucoxanthin; Hex: 19’-hexanoyloxyfucoxanthi; Pra: prasinoxanthin; Vio: violaxanthin; All: alloxanthin; Zea: zeaxanthin; Chlb: chlorophyll *b*; MVChla: monovinyl chlorophyll *a*; and DVChla: divinyl chlorophyll *a*.

### Conversion of MEX data to phytoplankton assemblage

The MEX excited the matter in the water using nine LED light sources (375, 400, 420, 435, 470, 505, 525, 570, and 590 nm), and the binary data recorded in the MEX were transformed into numerical values using the default MEX software (MFL software, JFE-Advantech, Hyogo, Japan). The MEXs were calibrated at the factory before shipping. The observations were conducted using three MEXs (MFL50W-USB, Serial No. 0042, 0052, and 0058). Thus, we evaluated the difference between the three MEXs at every excitation source by using ten types of algal culture collections (diatoms, cryptophytes, chlorophytes, and *Synechococcus*) in December 2019. The recorded fluorescence values were not significantly different among the sensors in every nine-light source (*Kruskal-Wallis* test, df = 2, chi-squared ≤ 0.34323, *p* ≥ 0.8423). Therefore, the difference of MEXs was not considered in this study.

The TChla concentration was estimated from MEX fluorescence and linear regression model: TChla concentration based on the MEX was abbreviated as TChla_MEX_. We used the “lmrob” function in package “robubase” [20] for the linear regression analysis. The intercept of regression analysis was set to zero to avoid the negative predicted TChla concentration. The coefficient between TChla and fluorescence was determined with M-regression estimation in “lmrob.” In addition to coefficient, outliers of the relationship can be detected in “robubase” [20]. The outliers were defined by weights as <10^−3^. The best fluorescence for estimation of TChla_MEX_ was determined with the coefficient of determination (*r*^2^).

Conversion from MEX fluorescence values to chemotaxonomic phytoplankton assemblages was performed using the linear inverse technique using the package “limSolve” [16] in R software [15]. We did not apply the Bayesian compositional linear inverse technique to save calculation resources.

We prepared a reference database (*U*) comprising MEX fluorescence (*F*_1_ –*F*_271_) and plant pigment-based chemotaxonomic phytoplankton assemblage (*P*_1_ –*P*_271_) data collected from the same water for conversion. The outliers (*o*) of the relationship between TChla and fluorescence were removed from the reference database. The MEX fluorescence (*F*_*i*_) was standardized to the sum of nine light-source emission fluorescence values of 1.


$$U=\{\left(F_{i},\;P_{i}\right)\;|\;1\leq i\leq 271,\;i⊄o\}.$$ (1)

Conversion of the observed MEX data (*f*) to the proportions of phytoplankton assemblages (*p*) was calculated in two steps using *U*. The observed MEX fluorescence for conversion (*f*) was then decomposed using the reference MEX fluorescence (*F*_1_ –*F*_271_) as the following equation:

$$f=\sum \alpha_{i}\times F_{i}$$ (2)

where the coefficient *α*_*i*_ was a number between 0 and 1, and the coefficients were established such that the residual was the lowest. We then calculated *p* (phytoplankton assemblage) using the coefficients (*α*_i_), as follows:

$$p=\sum \alpha_{i}\times P_{i}.$$ (3)

As a result, *f* was converted to *p*. The proportion of nine chemotaxonomy groups were detected using the plant pigment composition. Thus, we could calculate nine groups’ contributions by using these equations. The open-source R script, the reference dataset (*U*), and our MEX data were put in the repository (https://data.mendeley.com/datasets/5kzgcdf2kv).

### Validation and sensitivity analyses

We evaluated the effects of the fluorescence values of nine LEDs on plant pigment-based phytoplankton chemotaxonomy using permutational multivariate analysis of variance (PERMANOVA) in the “vegan” package [21] in R. The distances between samples were calculated based on the Bray–Curtis method, and the number of permutations was 1000. The fluorescence values’ effects on the phytoplankton community structure were checked using the determination coefficient (*R*^2^).

We evaluated the conversion process in Eqs (2) and (3). Here, two validation and one sensitivity experiments were conducted to evaluate the limitations of our conversion processes. The cross-validation approach was conducted. The target MEX fluorescence (*f*) was picked up from *F*, and the robustness was evaluated with the comparison between proportions of MEX-based phytoplankton assemblages (*p*) and plant pigment-based proportions (*P*). When either proportion or concentration of phytoplankton group (*m*) in *p* was not significantly correlated with those of *P*, we defined as the proportion of *m* cannot be evaluated using our conversion process. Based on the difference of proportions between *p* and *P*, we evaluated the confidence interval.

As the first validation experiment, we removed the samples collected at the same depth of the same station during the same cruise from *U*. The *p*_*j*_ was calculated from *F*_*j*_ based on the revised reference data set *U1*_*j*_. The *U1*_*j*_ was set as follows:

$${U1}_{j}=\{\left(F_{i},\;P_{i}\right)\;|\;1\leq i\leq 271,i⊄o,\;i\neq j\}.$$ (4)

The set of *p*_*j*_ based on *U1*_*j*_ was named as *p*_*test*_.

As the second validation experiment, we removed the samples collected from the same area (Kuroshio, JS, Okhotsk, and Oyashio). The *p*_*j*_ was calculated from *F*_*j*_ based on the reference data set *U2*_*j*_ set as follows:

$${U2}_{j}=\{\left(F_{i},\;P_{i}\right)\;|\;1\leq i\leq 271,\;{i⊄o,\;Area}_{i}\neq {Area}_{j}\},$$ (5)

where *Area*_*i*_ indicated the sampling area of data *i*. The set of *p*_*j*_ using *U2*_*j*_ was named *p*_*area*_.

As the sensitivity experiments, the bootstrap resampling (*R* = 1000) was applied to *U1*_*j*_ in (4), and then calculated *p*_*j*_. The set of *p*_*j*_ based on bootstrap resampling *U1*_*j*_ were named as *p*_*test_boot*_. Using *p*_*test_boot*_, the coefficient of variation (CV) of *p*_*j*_ were calculated in every phytoplankton group (CV_*jm*_). Then we calculated the mean and standard deviation of CV_*jm*_ in every phytoplankton group (CV_*m*_). When the CV_*m*_ was high, the conversion processes were primarily dependent on some of the data in *U*.

### Field MEX observations

The MEX was applied to the oceanic observations to show the spatial and seasonal variations of phytoplankton assemblages. The MEX observations were conducted at 366 stations during 23 cruises around Japan in 2016, 2019, and 2020; however, we focused on the observations at the four stations where repeated observations were conducted (Fig 1): stn. A01 (42.83°N, 144.83°E) in the Oyashio, stn. C3400 (34°N, 138°E) in the Kuroshio, stn. N4 in the Okhotsk (45.41°N, 145.17°E), and stn. SI09 (41°N, 136.34°E) in the JS. The observations were conducted four, five, three, and six times at stn. A01, C3400, N4, and SI09, respectively.

Fluorescence measurements were obtained by the MEX, recording the temperature and pressure (depth of the sensor). The observations were conducted regardless of the time; the sensors’ detector was set downward and placed where prevented the reflections of the LED or sunlight. Values were recorded every 0.1 s. To remove the spike values, we calculated median values of every 1 s, which were applied to identify the vertical distribution of the phytoplankton community structure. MEX starts recording the data at a pre-programmed time and continuously records until the connection to a personal computer. We sometimes continuously used MEX for several stations without turning off its power, and the profiles at several stations were recorded in one binary file. To pull out a single station data set form those of plural stations, data from a sensor depth of ≥2 m over 300 s were categorized as “observations”, and identified as every observation. Therefore, the MEX-based results at the surface indicated those at 2-m depth. The start timing and numbers of the observations based on this definition were consistent with those recorded in field books, and observation positions was obtained from the field books. We converted the 1-s MEX data to 1-m binned data using the pressure data of the MEX. The observations were conducted from the depths of 2 m to 100 m at stn. A01 and N4, to 150 m at stn. SI09, and to 200 m at stn. C3400.

The vertical heterogeneity was evaluated with a quartile coefficient of dispersion (QCoD) based on proportions’ first and third quartiles. The water-column integrated concentration of every phytoplankton group at every station and observation was calculated based on the proportion and TChla_MEX_. Similarities of station and observation were evaluated by using a non-metric multidimensional scaling technique (NMDS). The distances among the samples were calculated based on the Bray-Curtis dissimilarity.

## Results

### Estimation of TChla concentration

The TChla (MVChla + DVChla) was most strongly correlated with fluorescence excited at 435 nm (*F435nm*, *r*^2^ = 0.8844, *n* = 271), followed by that at 470 nm (*F470nm*, *r*^2^ = 0.8644, *n* = 271). The coefficient values of the linear model between TChla and *F435nm* was 0.54095 (standard error: 0.02205). Thus, we estimated TChla_MEX_ concentration with the following equation.


$${TChla}_{MEX}=0.54095\;\times \;F435nm.$$ (6)

In the linear model between TChla and *F435nm*, 23 data points were categorized as the outliers, and those outliers were removed from the reference database (*U*). As the result, the numbers of reference database (*U*) were 248 in the following analyses.

### Validation of conversions

PERMANOVA indicated the nine light-source-excited fluorescence measurements explained the significant differences observed in the chemotaxomic phytoplankton groups (*p* < 0.05, *n* = 248). The detection coefficient of every light source differed among the equations, and the sum of the detection coefficients was 0.328 (residuals *r*^2^ = 0.672).

**Fig 2:**
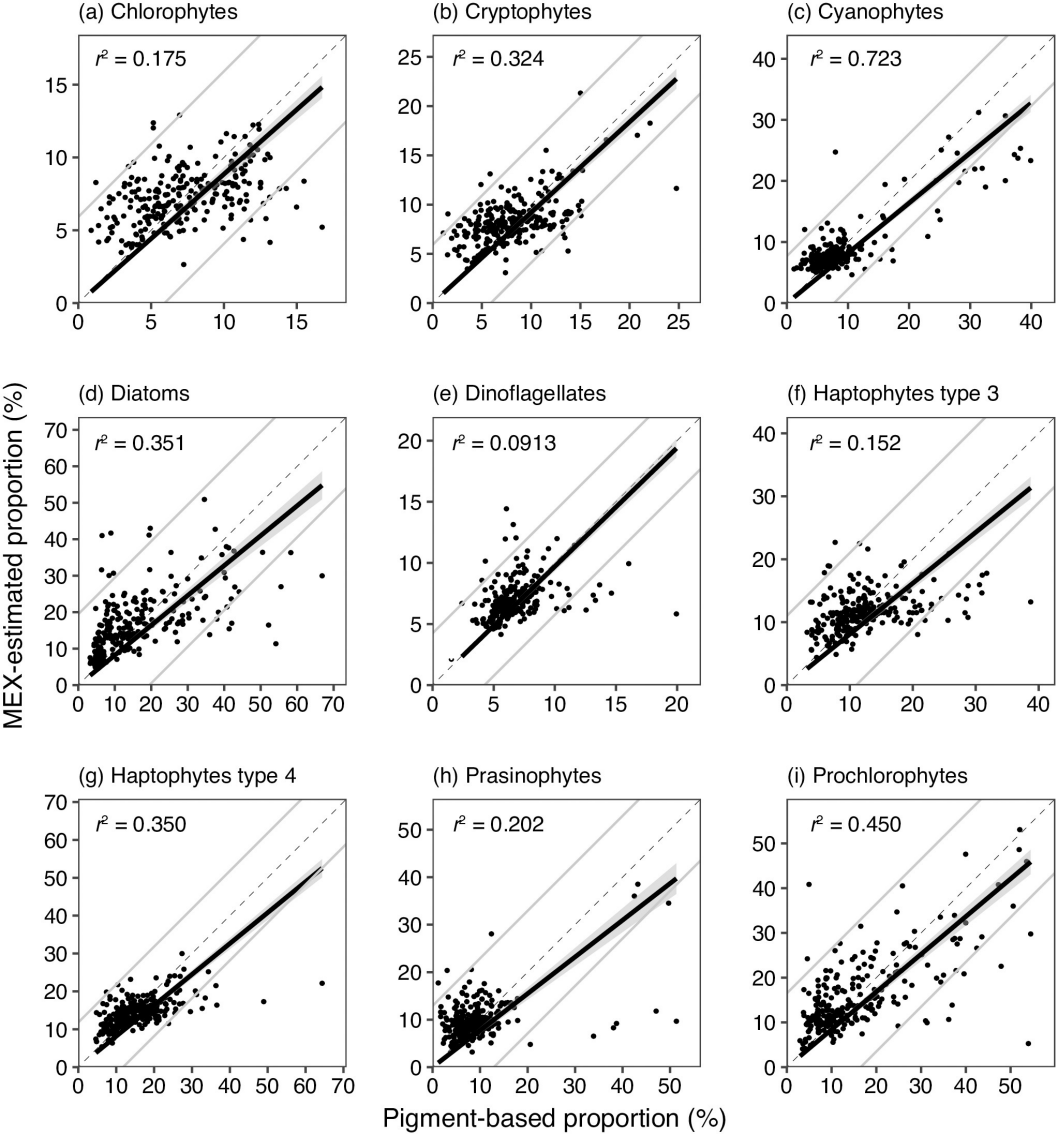
Scatter plots between MEX-estimated phytoplankton proportions (%) to TChla_MEX_ concentration and pigment-based phytoplankton proportions (%) to TChla concentration. MEX, TChla_MEX_ TChla are abbreviations of *in situ* multi-wavelength excitation fluorometer, total chlorophyll *a* concentration estimated with MEX and Eq (6), total chlorophyll *a* concentration estimated with the pigment analyses. Comparisons were performed in nine chemotaxonomic phytoplankton groups (chlorophytes, cryptophytes, cyanophytes, diatoms, dinoflagellates, haptophyte type 3 haptophyte type 4, prasinophytes, and prochlorophytes). The MEX-based proportion was estimated based on the database (*U*1), which only removed the target data from *U*. The thin dased and the bold lines denote the 1:1 line and the linear regression line, respectively. The gray lines indicated 95% confident intervals of the differences between MEX-based and pigment-based proportions.

**Fig 3:**
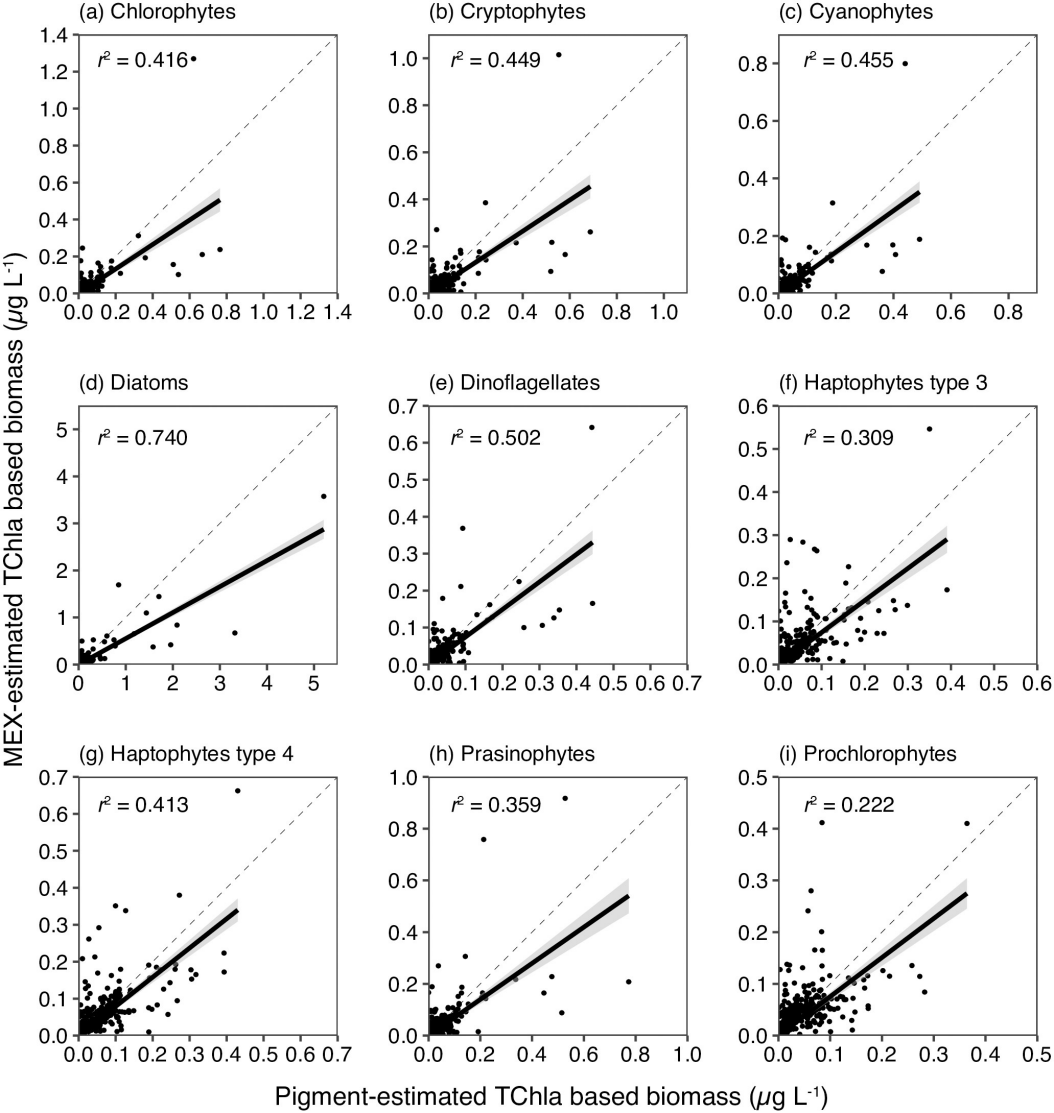
Comparisons of MEX estimated and pigment-based of chlorophyll-*a* concentration (μg L^-1^). Comparisons in nine chemotaxonomy phytoplankton groups (chlorophytes, cryptophytes, cyanophytes, diatoms, dinoflagellates, haptophyte types 3 and 4, prasinophytes, and prochlorophytes). The concentration was computed by multiplying proportions of phytoplankton by TChla or TChla_MEX_. The thin dashed line denotes the 1:1 line, and the bold line with shadow denotes the linear regression line.

At first, *p*_*test*_ was compared with *P*. The significant positive relationships between *p*_*test*_ and P proportions were observed in all the nine phytoplankton groups (each *n =* 248, *p* < 0.001, *t*-value >24.2, Fig 2). The difference between proportions in *p*_test_ and those in *P* was largest in diatoms (mean absolute difference was 6.68%) in nine chemotaxonomy groups. The second largest was observed in prochlorophytes (mean absolute difference was 5.66%). The 95% confidence intervals of the differences indicated the proportion of diatoms using the MEX could be estimated ±19.5% from the plant-pigment based one, and those of prochlorophytes were ±16.5%. The other plankton groups were <±12.9%. The chlorophyll-*a* based biomass of phytoplankton groups obtained for the products of *p*_*test*_ and TChla_MEX_ were also significantly correlated with pigment-based biomass obtained for the products of *P* and TChla concentration (each *n =* 248, *p* < 0.001, Fig 3).

**Fig 4:**
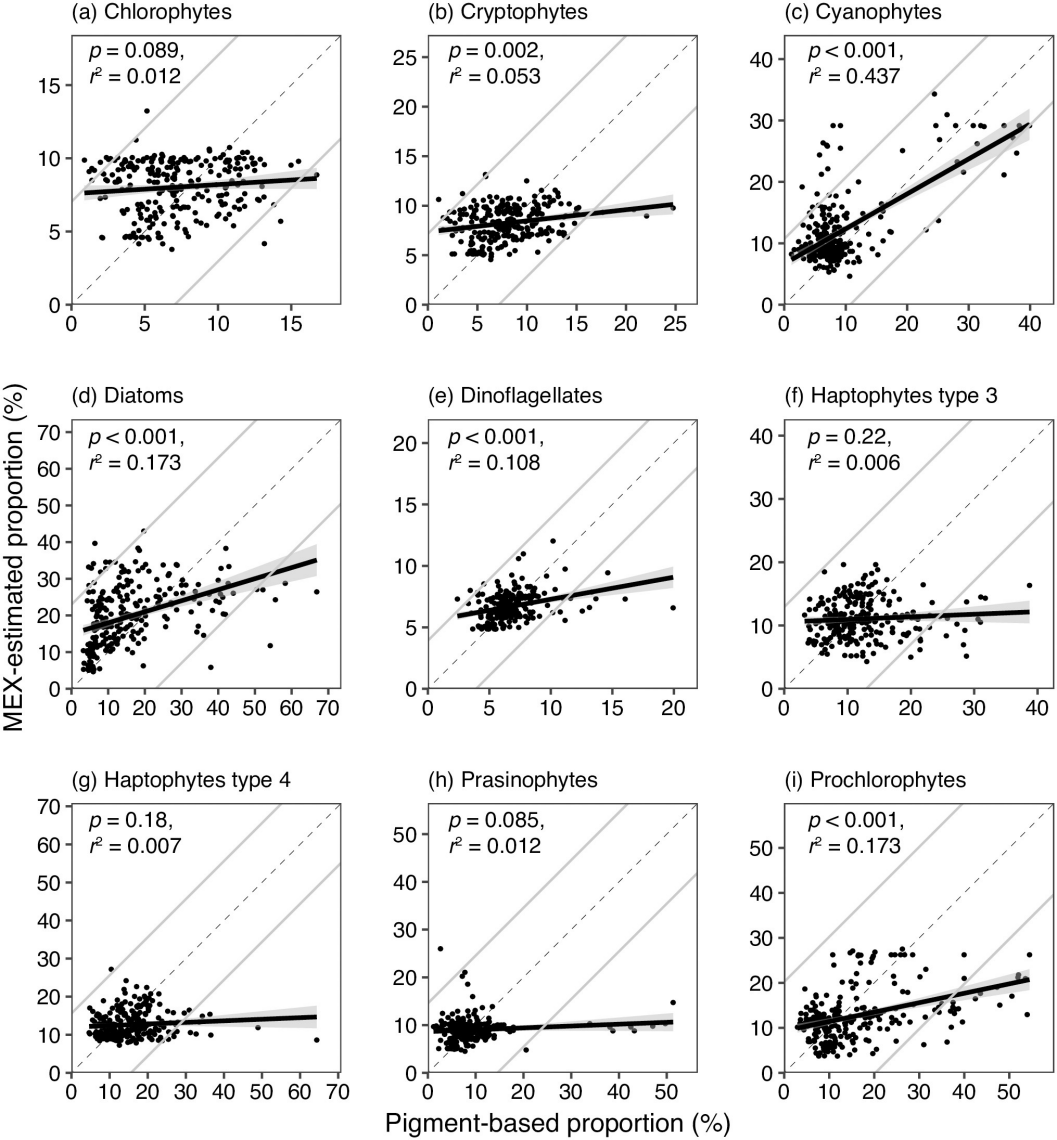
Same scatter plots with Fig 2, but MEX-estimated phytoplankton proportions (%) were based on the database (*U*2), which removed the data collected in the same area from *U*. Comparisons were performed in nine chemotaxonomic phytoplankton groups (chlorophytes, cryptophytes, cyanophytes, diatoms, dinoflagellates, haptophyte type 3, haptophyte type 4, prasinophytes, and prochlorophytes). The dashed and the bold lines with shadow denote the 1:1 line and the linear regression line with standard errors regression analysis, respectively. The gray lines indicated 95% confident intervals of the differences between MEX-based and plant pigment-based proportions.

**Fig 5:**
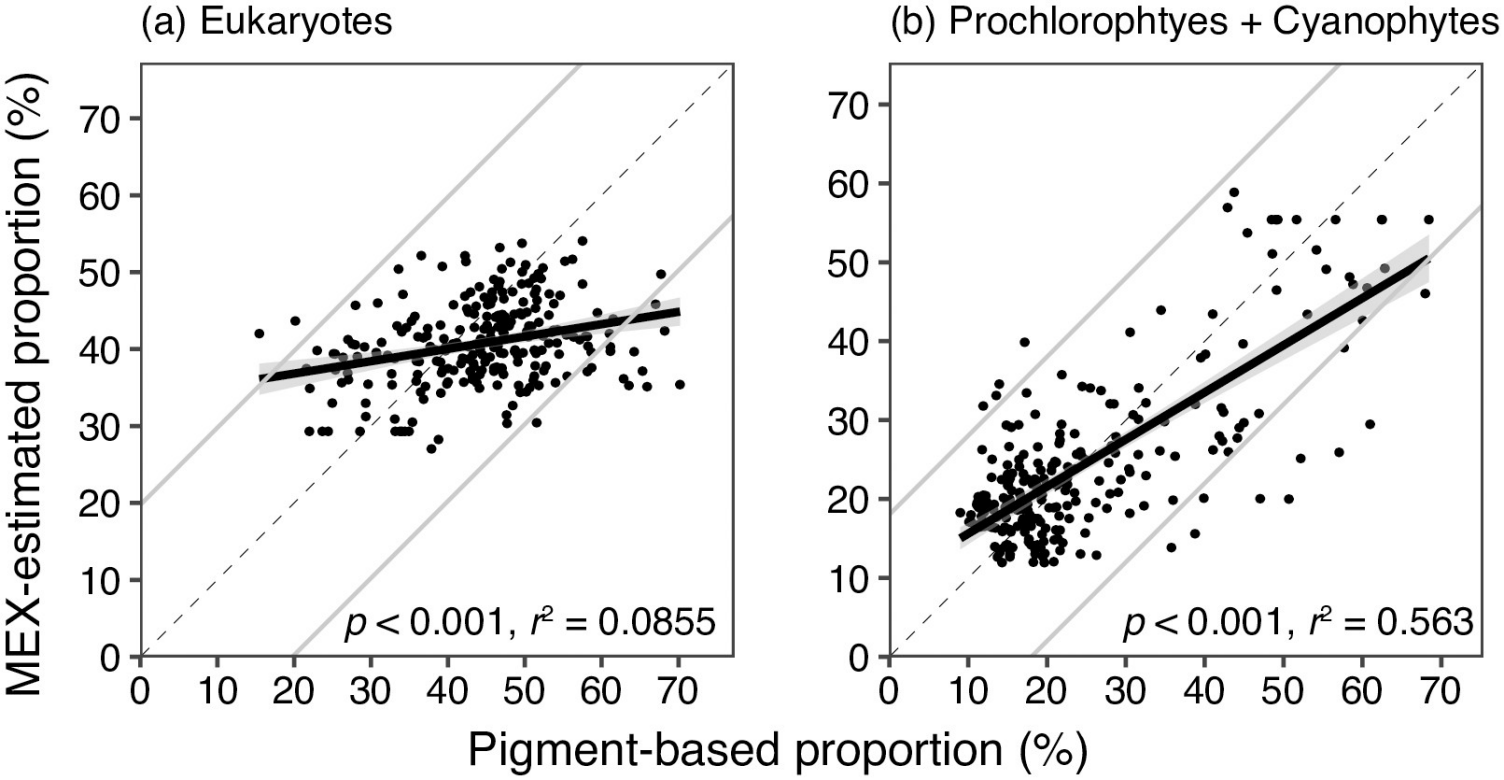
Same scatter plots with Fig 4 but performed in (a) eukaryotes and (b) sum of cyanophytes and prochlorophytes to TChla (total chlorophyll *a*) concentration. The MEX-based proportion was estimated based on the database (*U*2), which removed the data collected in the same area from *U*. The dashed and the bold lines with shadow denote the 1:1 line and the linear regression line with standard errors regression analysis, respectively. The gray lines indicated 95% confident intervals of the differences between MEX-based and plant pigment-based proportions.

Compared between *p*_*area*_ and *P*, no significant relationships were observed in the case of chlorophytes, haptophytes type 3, haptophytes type 4, and prasinophytes (*n* = 248, *p* > 0.05, *t*-test, Fig 4). The significant positive relationships were observed in the case of other five phytoplankton groups: cryptophytes, cyanophytes, diatoms, dinoflagellates, and prochlorophytes (*n* = 248, *t*-test, *p* < 0.01). When chlorophytes, prasinophytes, haptophytes types 3 and 4 were summarized and named as eukaryotes, the MEX-estimated eukaryotes proportions were positively correlated with those estimated by plant pigment composition (*n* = 248, *t*-test, *p* < 0.001, *r*^2^ = 0.0855, Fig 5). We also calculated the sum of prochlorophytes and cyanophytes, and significant positive relationships were observed between the MEX-estimated proportion and plant pigment-based proportion (*n* = 248, *t*-test, *p* < 0.001, *r*^2^ = 0.563, Fig 5).

### Sensitivity analyses

**Fig 6:**
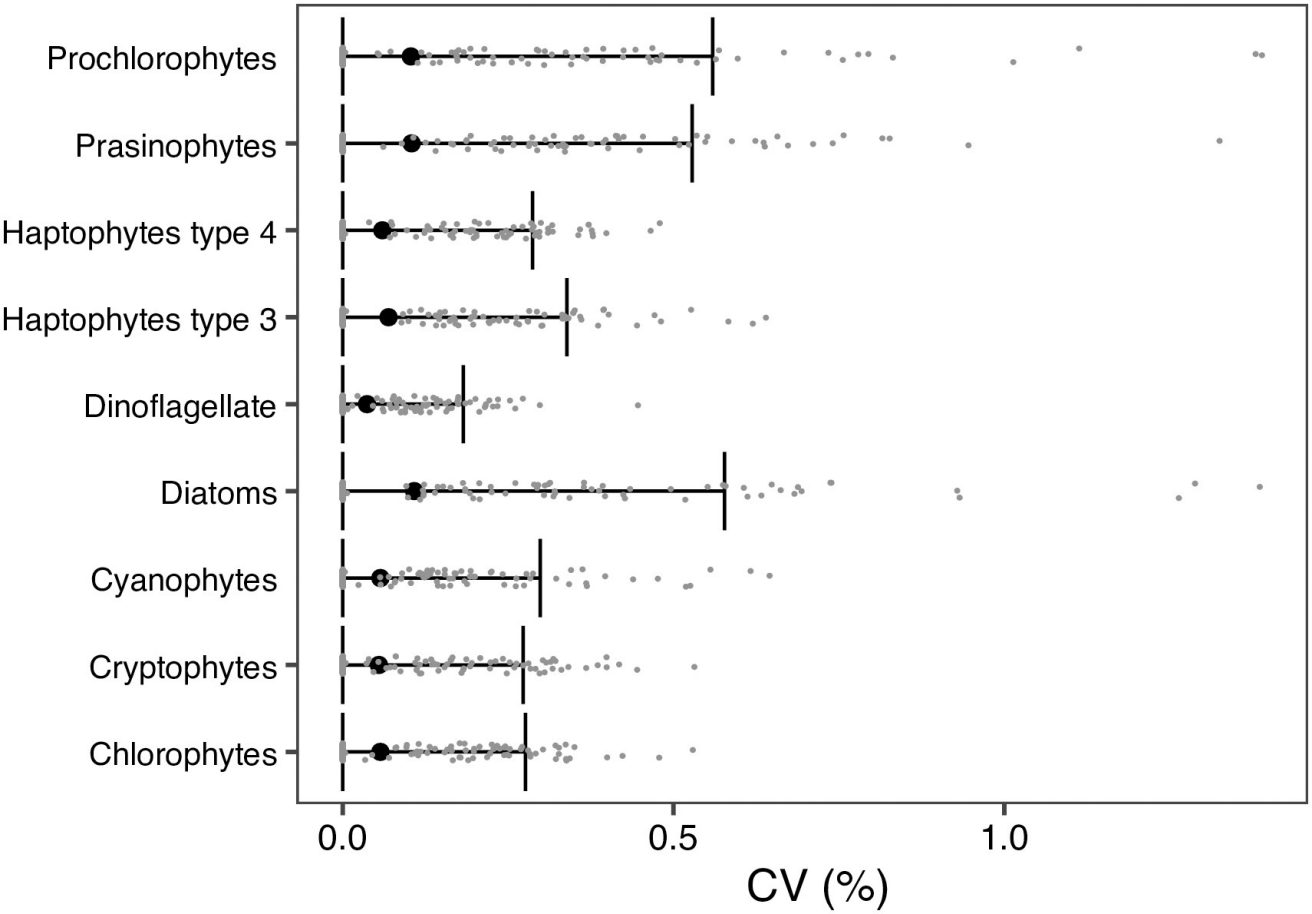
Variations of CV_*m*_ (%) based on the bootstrap resampling of the reference database. The black circles with whiskers indicated mean values with 95% confident intervals of CV_*m*_. The small gray point indicated the coefficient of variation (CV) of the proportion of one target data set calculated based on the bootstrap resampling database from the reference database. Still, the target data was permanently removed (*U*1).

Based on *p*_*test_boot*_, the 95% confidence intervals (mean ± 2 sd) indicated that the CV*m* values were very low: they varied <1% (Fig 6). The upper limits of 95% confident intervals of CV_*diatom*_ (CV values of diatom), CV_*prasinophytes*,_ and CV_*prochlorophytes*_ were 0.577%, 0.528%, and 0.559%, respectively. Those of the other 6 groups were <0.4%.

### Field applications of MEX

**Fig 7:**
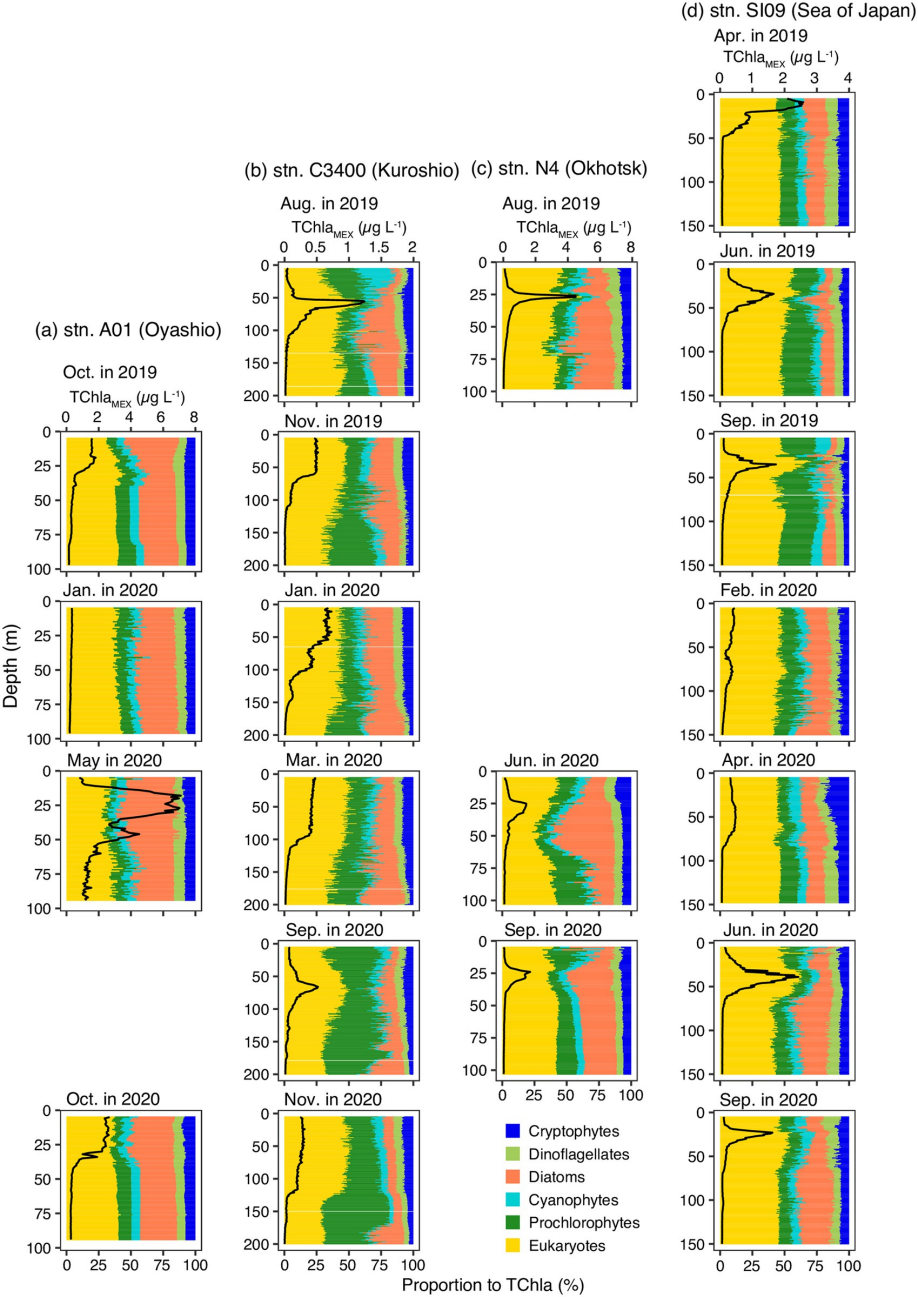
Vertical distributions of MEX (Multi-Exciter)-based chemotaxonomic proportions and TChla concentration. Distributions at (a) stn. A01 in the Oyashio, (b) stn. C3400 in the Kuroshio, (c) stn. N4 in the Okhotsk Sea, and (d) stn. SI09 in the Sea of Japan (JS). The solid black line represents the MEX-based Tchla concentration (Tchla_MEX_). The scales of Tchla_MEX_ were the same in every area regardless of the season. The panels were sequenced from spring in 2019 to autumn in 2020. The blank panels indicated that the observations were not conducted at that station in that season and year. At the stn. SI09, vertical profiles were observed twice in the summer.

**Fig 8:**
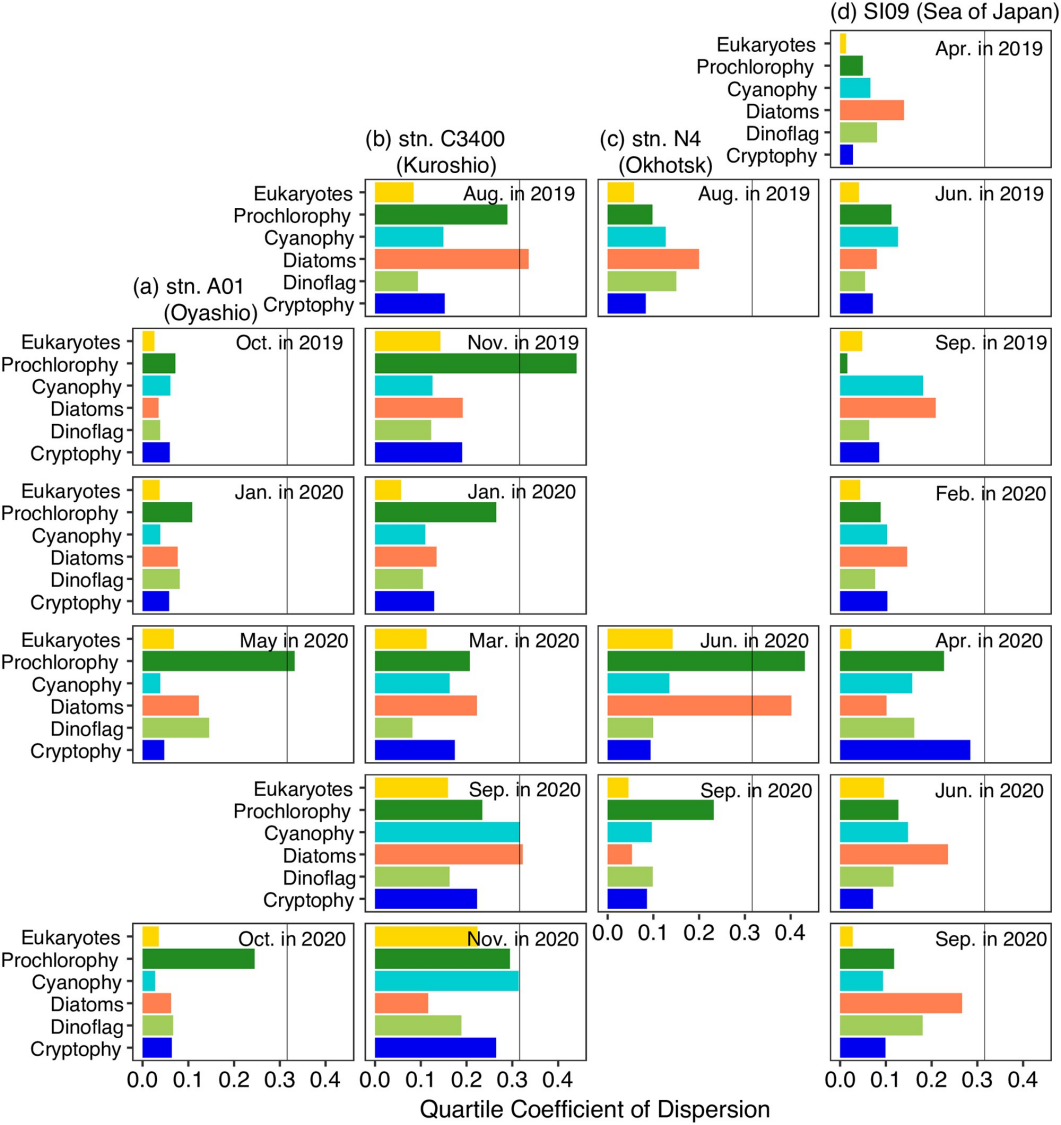
Vertical heterogeneity indices of phytoplankton assemblages based on quartile coefficient of dispersion (QcoD). The QcoD values of phytoplankton chemotaxnomy (a) stn. A01 in the Oyashio, (b) stn. C3400 in the Kuroshio, (c) stn. N4 in the Okhotsk Sea, and (d) stn. SI09 in the Sea of Japan (JS). The solid black vertical line represents the threshold of high heterogeneity (mean ± 2 sd of QcoD) of the vertical profiles.

The MEX observations at four stations showed significant temporal and area differences (Fig 7). The vertical heterogeneity was evaluated with the QCoD of every profile and phytoplankton group (Fig 8). The mean ± sd of QCoD based on every profile and phytoplankton group was 0.134 ± 0.0912 (*n* = 120): we set 0.316 (mean ± 2 sd) as the threshold of high heterogeneity of the vertical profiles. As a result, high heterogeneity of the vertical profiles of diatoms was observed at stn. C3400 in August of 2019 and September of 2020 (Fig 8b), and at stn. N4 in June of 2020 (Fig 8c). High heterogeneity of prochlorophytes proportion was observed at stn. A01 in May of 2020 (Fig 8a), at stn. C3400 in the November of 2019 (Fig 8b), and at stn. N4 in June of 2020 (Fig 8c). High heterogeneity of cyanophytes proportions was only found at stn. C3400 in September of 2020 (Fig 8b). The diatoms’ proportion was high, just below the subsurface chlorophyll maximum at stn. C3400 in August of 2019 (Fig 7b) and at stn. N4 in June of 2020 (Fig 7c). On the other hand, diatoms contribution is high in the ≥175 m at stn. C3400 in September of 2020 (Fig 7b). The proportion of prochlorophytes at stn. A01 in May of 2020 was low (median:7.3%), and varied unstably at this station (Fig 7a).

**Fig 9:**
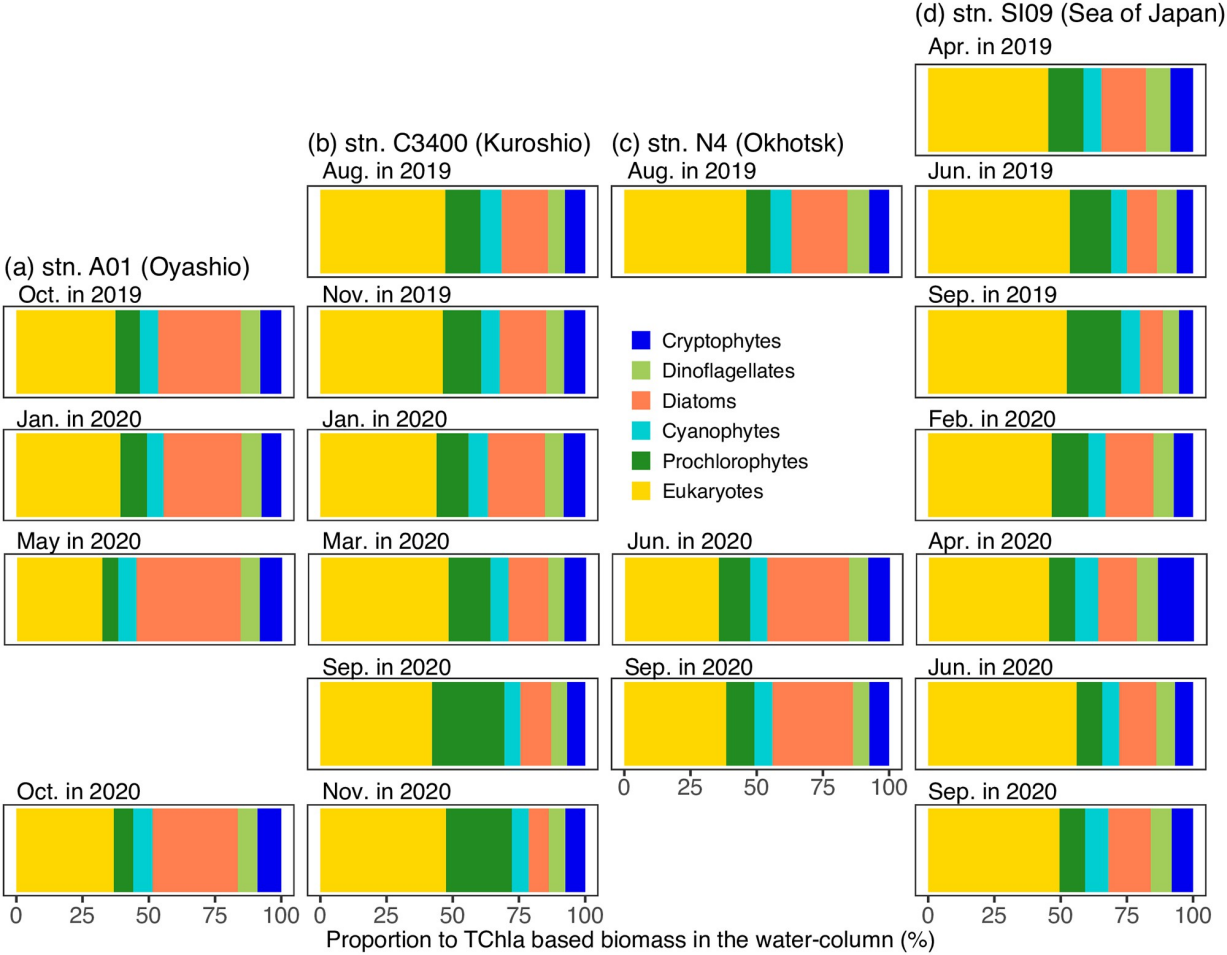
Temporal and spatial variations of proportion to Tchla-based biomass in the water column. The water-column integrated Tchla-biomass was calculated (a) from 2 to 100 m depth at stn. A01 in the Oyashio, (b) from 2 to 200 m depth at stn. C3400 in the Kuroshio, (c) from 2 to 100 m depth at stn. N4 in the Okhotsk Sea, and (d) at from 2 to 150 m depth stn. SI09 in the Sea of Japan (JS).

The water-column integrated TChla based biomass of every phytoplankton group at every station and depth indicated that the eukaryote was dominant in the water column at all the stations except at stn. A01 in May of 2020, where eukaryote was second dominant, and diatoms were dominant (Fig 9). The second dominant phytoplankton groups were diatoms or prochlorophytes. The diatoms were the second dominant group in all the observations except in May of 2020 at stn. A01 (Fig 9a), in August of 2019, November of 2019, and January of 2020 at stn. C3400 (Fig 9b), in all the observations at stn. N4 (Fig 9c), and in all the observations except in June and September of 2019 at stn. SI09 (Fig 9d). At the other observations, prochlorophytes were the second dominant group (Fig 9).

**Fig 10:**
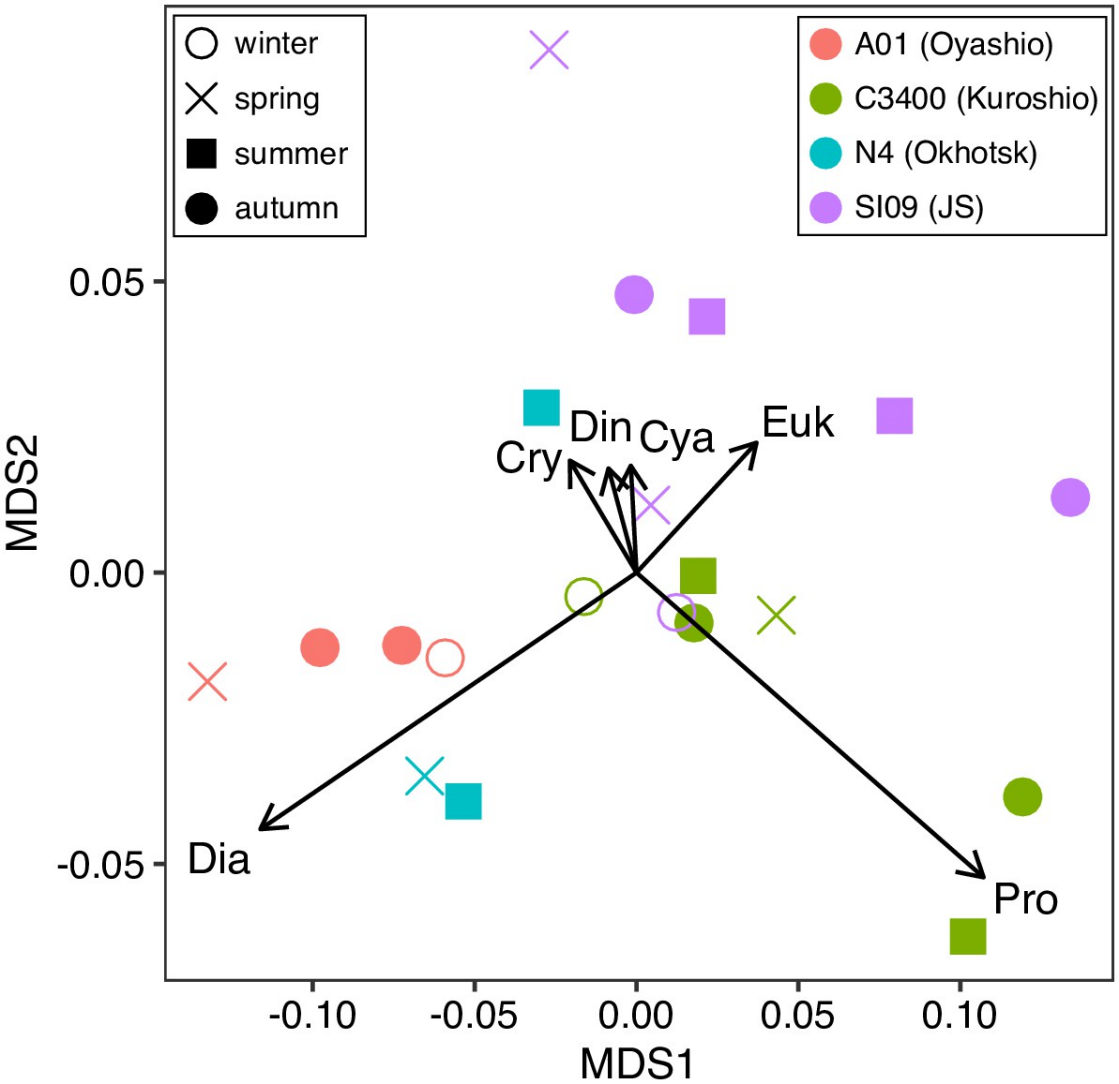
Similarities of proportion to TChla-based biomass in the water column among the observations. Similarities were calculated on the Bray-Curtis index and the non-metric multidimensional scaling (nMDS). The shapes and colors of the symbols indicated the sampling season and stations, respectively. The arrows with text denote the effect of phytoplankton chemotaxonomy groups (Cry: Cryptophytes, Euk: Eukaryotes, Pro: Prochlorophytes, Cya: Cyanophytes, Dia: Diatoms, and Din: Dinoflagellates).

On the basis of the water-column integrated TChla based biomass of every phytoplankton group at every station and depth, the similarities of the observations were calculated. The stress of the NMDS was 0.066. The NMDS approach showed that observations at the same stations are plotted in similar places compared to the same seasons (Fig 10). The observations at stn SI09 in the JS were plotted in the first quadrant, those at stn. C3400 in the Kuroshio were plotted in the second quadrant, and those at stn. A01 in the Oyashio were plotted in the third quadrant (Fig 10). The first, second and third quadrants were characterized by high proportions of eukaryotes, prochlorophytes and diatoms, respectively.

## Discussion

Multi-Exciter (MEX) data only explained 32.8% of plant pigment-based chemotaxonomic data based on PERMANOVA, suggesting that the MEX observations theoretically explained one-third of the variations in the phytoplankton assemblage. However, our empirical conversion methods enabled the evaluation of six chlorophyll-*a*-based chemotaxonomic groups based on the cross-validation analyses. The six chemotaxonomic phytoplankton groups are greater than those previously reported [7, 8, 10]. In the previous studies [7, 8, 10], the sensors can measure the biomass of four plankton groups (green algae, brown algae, cyanobacteria and mixed). Specifically, our method distinguished the contributions of diatoms and dinoflagellates. Yentsch and Yentsch [22] showed that the excitation spectra of diatoms and dinoflagellates are similar. However, Johnsen et al. [23] showed that the peridinin–chlorophyll *a*-binding protein complex demonstrated a high degree of fluorescence when excited at between 480 nm and 510 nm (peridinin is a marker pigment of dinoflagellates [1]). Because the MEX at an excitation wavelength of 505 nm using an LED, the contributions of diatoms and dinoflagellates could be distinguished. We confirmed that the ratio of *F505nm*:*F435nm* had a significant positive relationship with proportion of dinoflagellates (*n* = 248, *t*-value = 3.224, *p* = 0.00144). However, some dinoflagellates did not have peridinin [24], and dinoflagellates lacking peridinin cannot detect as dinoflagellates by the CHEMTAX approach without their unique pigments [25]. We did not have microscopic observations in this study, and thus the dinoflagellates abundance may be underestimated if there are peridinin-lacking dinoflagellates in our observations.

On the other hand, we cannot find any theoretical background to identify the contributions of cyanobacteria and prochlorophytes separately. The prochlorophytes biomass is low in the cold waters [26], but our MEX observation identified a significant contribution of prochlorophytes at stn. A01 (Oyashio area) in winter. In addition, the LEDs of MEX have detected the emission of phycoerythrin and phycocyanin [9, 27]. The ratios of phycoerythrin: TChla and phycocyanin: TChla broadly vary with environmental conditions [27, 28], and fluorescent signals of *Synechococcus* (major cyanophytes) and prochlorophytes were very similar [29]. These indicated the MEX approach could not divide the contribution of prochlorophytes and cyanophytes. The cross-validation analyses observed positive relationships between MEX-based and pigment-based proportions to TChla in the cyanophytes and prochlorophytes, but we considered our identified proportions of prochlorophytes and cyanophytes should be summed and treated as cyanobacteria.

The empirical analyses usually depend on the reference data, particularly the size of the reference data. Wang et al. [10] reported that 26 samples are generally sufficient for their model calibration: the method is different, but our reference data set (*n* = 248) is approximately ten times higher than the sufficient numbers. Our sensitivity analysis supports this: the proportion of phytoplankton largely varied only <1% when the reference data were randomly selected (Fig 6). Therefore, we considered our reference data set robust for evaluating phytoplankton assemblages in the western North Pacific.

Our present study is the first report on water-column-integrated phytoplankton biomass at the chemotaxonomic group level in the Pacific Ocean. MEX allows identification of fine-scale vertical variations of phytoplankton assemblages as Figs 7 and 8, and calculation of the water-column-integrated biomass of every group as Fig 9. Bracher et al. [30] first estimated the water-column-integrated biomass using hyperspectral underwater-irradiance radiometer data in the Atlantic Ocean. These radiometers can only be applied during the day, which suggests an advantage for the MEX. The MEX observations can be conducted regardless of time.

The phytoplankton assemblages in our study depended on the pigment-based estimation, and the initial ratio (Table 2) should be more area- and season-specific ones to identify the spatial difference of phytoplankton assemblages more accurately. The field application of MEX, however, clearly showed that phytoplankton assemblages are different among the regions (Fig 10). In the JS, the eukaryotes (chlorophytes, prasinophytes, haptophytes), which consider comprising pico- and nano-sized phytoplankton, were abundant in every season, and higher contribution of eukaryotes to the TChl-based biomass was the unique feature compared to the other three areas (Fig 10). In particular, the contributions of eukaryotes to the TChla concentration were large at the subsurface chlorophyll maximum. This is different from the cases in the Okhotsk and the Oyashio areas. In the Okhotsk and the Oyashio, the diatom contribution was high, which agreed with the previous reports [31–33], and suggested that the silicate uptake is active in the Okhotsk and the Oyashio compared to the JS and Kuroshio area [34]. In the Kuroshio area, the contribution of eukaryotes to the TChl-based biomass was high, but the high contribution of cyanobacteria (cyanophytes and prochlorophytes) was the unique feature of this area. In particular, the high contribution of cyanobacteria was detected in the Kuroshio area during summer near the surface, and this was the same as that in the more coastal and offshore areas under the influence of the Kuroshio area [18, 35].

Our vertical profile of phytoplankton assemblages indicated that the phytoplankton community varies around the subsurface chlorophyll maximum as pointed by Latasa et al. [36]. The contributions of the phytoplankton chemotaxonomic groups were not homogenous in vertical not only at the subsurface chlorophyll maximum. For example, highly vertical heterogeneity was found at stn. N4 in June. In this observation, eukaryote proportion was high above the subsurface chlorophyll maximum, diatom proportion was high just above the subsurface chlorophyll maximum, and then cyanobacteria contribution was high below 75 m depth. Hirata et al. [4] reported surface phytoplankton biomass at the chemotaxonomic group level based on space-borne ocean-color sensors. Uitz et al. [37] estimated the biomass of microplankton, nanoplankton, and picoplankton based on the surface chlorophyll-*a* concentration. We did not have any results on the size-fractionation phytoplankton abundance. Assuming cyanophytes and prochlorophytes are mainly categorized as picoplankton and diatoms are as microplankton, our MEX observations suggested that not only assemblages but also size fractionation are heterogeneous in vertical. Therefore, only surface observations provide very limited knowledge of ocean productivity, and the combinations of surface observations and MEX observations are necessary for understanding the three-dimensional (longitude, latitude and depth) or four-dimensional (longitude, latitude, depth and time) variations of ocean productivity.

## Conclusion

In this study, we described a method allowing reliable estimation of phytoplankton-community structure using the MEX. The two validation analyses and one sensitivity analysis indicated that our conversion method is robust, and well estimated the proportions of six phytoplankton chemotaxonomy groups––namely, diatoms, dinoflagellates, cryptophytes, cyanophytes, prochlorophytes, and other eukaryotes (chlorophytes, haptophytes, and prasinophytes)––to TChla (total chlorophyll *a*) concentration. The field application indicated that the vertical distributions of phytoplankton assemblages are heterogenous, and water-column integrated compositions of the phytoplankton community showed the spatial features in the oceans around Japan. These suggested that MEX is a powerful tool for understanding ocean productivities. We considered that our approach represents a promising method for potential worldwide observations. Our database and computer program are opened, and the program is written by open-source R language, and thus our methods help understand the phytoplankton assemblages in the world ocean. The primary productivity is decreasing with the global warming everywhere [38], but we don’t have little knowledge on the changes of the phytoplankton community. The MEX observations will be a promising solution to understand the changes of the phytoplankton community in the global ocean.
